# Phosphorylation of the Regulators, a Complex Facet of NF-κB Signaling in Cancer

**DOI:** 10.3390/biom11010015

**Published:** 2020-12-26

**Authors:** Aishat Motolani, Matthew Martin, Mengyao Sun, Tao Lu

**Affiliations:** 1 Department of Pharmacology & Toxicology, Indiana University School of Medicine, Indianapolis, IN 46202, USA; amotolan@iu.edu (A.M.); mm217@iu.edu (M.M.); sun19@iu.edu (M.S.); 2 Department of Biochemistry & Molecular Biology, Indiana University School of Medicine, Indianapolis, IN 46202, USA; 3 Department of Medical & Molecular Genetics, Indiana University School of Medicine, Indianapolis, IN 46202, USA

**Keywords:** cancer signaling, NF-κB, phosphorylation, PRMT5, YBX-1

## Abstract

The nuclear factor kappa B (NF-κB) is a ubiquitous transcription factor central to inflammation and various malignant diseases in humans. The regulation of NF-κB can be influenced by a myriad of post-translational modifications (PTMs), including phosphorylation, one of the most popular PTM formats in NF-κB signaling. The regulation by phosphorylation modification is not limited to NF-κB subunits, but it also encompasses the diverse regulators of NF-κB signaling. The differential site-specific phosphorylation of NF-κB itself or some NF-κB regulators can result in dysregulated NF-κB signaling, often culminating in events that induce cancer progression and other hyper NF-κB related diseases, such as inflammation, cardiovascular diseases, diabetes, as well as neurodegenerative diseases, etc. In this review, we discuss the regulatory role of phosphorylation in NF-κB signaling and the mechanisms through which they aid cancer progression. Additionally, we highlight some of the known and novel NF-κB regulators that are frequently subjected to phosphorylation. Finally, we provide some future perspectives in terms of drug development to target kinases that regulate NF-κB signaling for cancer therapeutic purposes.

## 1. Introduction

### 1.1. Brief Overview of Cancer and Key Signaling Pathways

Cancer is a diverse and multifactorial genetic disease that arises through a multistep accumulation of genetic alterations, which causes genomic instability in a cell. This genomic instability results in aberrant cellular functions, such as uncontrolled growth, cell death resistance, increased cell migration and invasion, evasion of immune surveillance, metabolic reprograming, etc. [1]. The progression of cancer is further driven by the complex interaction of malignant cells with neighboring cells in their microenvironment [2].

Genetic mutations in multiple signaling pathways have been linked to cancer progression. Several typical examples include the receptor tyrosine kinase/Ki-ras2 Kirsten rat sarcoma viral oncogene homolog (RTK/KRAS) pathway, tumor protein P53 (p53) pathway, transforming growth factor β (TGFβ) pathway, and phosphoinositide 3-kinase/Akt (PI-3-kinase/Akt) pathway, etc. Interestingly, ample evidences suggest that these signaling pathways frequently promote cancer progression through the nuclear factor κB (NF-κB) activation [3,4]. For example, KRAS oncogenic mutation and p53 loss of function mutation, which is present in approximately 25% and 50% of human tumors, respectively, leads to the constitutive activation of NF-κB, thereby promoting cell survival in multiple cancers. [4]. Similarly, mutations in the PI-3-Kinase/Akt pathway, which exist in over 30% of solid tumors, promotes the activation of components in NF-κB pathway [2,5]. These examples, among many others, demonstrate the complex signaling interactions in cancer and the pivotal role that NF-κB plays in enabling cancer progression. Hence, it is of great clinical importance to fully understand the different facets of NF-κB regulation in cancer. In this review, we will further elaborate on the complexity of NF-κB regulation in cancer, with the goal of providing deep insight and aiding the development strategies of novel NF-κB targeted cancer therapeutics. 

### 1.2. Overview of NF-κB Signaling 

**Figure 1:**
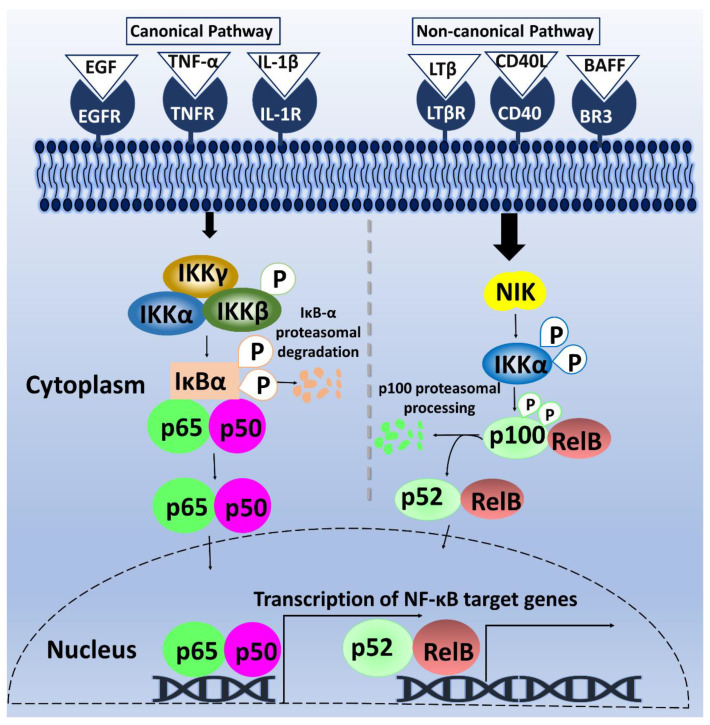
Schematic diagram of NF-κB canonical and non-canonical pathways. In the canonical pathway, on the left, the stimulation of receptors by cytokine and growth factors lead to the phosphorylation of IKK complex, which in turn phosphorylates IκBα, leading to its proteasomal degradation. The free p65/p50 dimers of NF-κB then translocate into the nucleus to bind to κB motif and promote NF-κB-dependent gene transcription. On the right, the non-canonical pathway is activated by binding of LTβ, CD40L, and B-cell activating factor (BAFF) to their respective receptors, causing NIK-mediated phosphorylation of IKKα. Phosphorylation of p100 by activated IKKα initiates proteasomal processing of p100 N-terminal to form p52. The p52/RelB dimeric complex then undergoes nuclear translocation and binds to its κB motif, resulting in NF-κB-dependent gene transcription.

Gene transcription plays a fundamental role in mediating several biological processes. Thus, strict regulation of transcription factors is necessary to maintain cellular homeostasis. One such transcription factor is NF-κB. The omnipresent NF-κB is a group of homo- and hetero-dimeric proteins, which was discovered about three decades ago in B lymphocytes. NF-κB was found to bind to a B motif at the enhancer element of the κ light-chain gene to regulate its expression [6,7]. The κB motif, as it is currently termed, consists of the following sequence: 5′-GGGRNNYYCC-3′; wherein Y = pyrimidine, R = purine, and N, any nucleotide. After years of continuous studies on NF-κB, additional mechanistic insights into the roles of NF-κB and its signaling cascade—beyond B-cells—have been elucidated [8]. Mammalian NF-κB is composed of five-member subunits that dimerize at gene promoters to control differential gene expression. The members of the NF-κB family include p65/RelA, RelB, c-Rel, p50/p105 (NF-κB1), and p52/p100 (NF-κB2) [9]. These subunits are characterized by the Rel-homology domains in their N-terminal, which contains a DNA-binding domain, a nuclear localization sequence, and a dimerization domain [8]. Additionally, p65, RelB, and c-Rel contain a transactivation domain in their C-terminal, enabling their transcriptional activity. In contrast, the C-terminal region of p105 and p100, the precursors of p50 and p52 respectively, lacks a transactivation domain but contains several ankyrin repeat sequences that function to inhibit NF-κB [10]. As shown in Figure 1, the mechanism of NF-κB induction is grouped into two pathways: canonical and non-canonical pathways. In the canonical pathway, external stimuli such as growth factors and cytokines bind to NF-κB cell-surface receptors to activate the phosphorylation of Inhibitor of κB (IκB) by Inhibitor of κB kinase (IKK). This phosphorylation results in IκBα degradation, causing the translocation of p65/p50, c-Rel/p50, or p65/p52 dimers to the nucleus. These dimers bind to their respective κB elements on the genes [11,12,13] (Figure 1). Comparably, the non-canonical pathway involves signaling *via* Cluster of differentiation 40 (CD40) receptor, Lymphotoxin-β receptor (LTβR), and BLyS receptor 3 (BR3) receptors, triggering NF-κB -inducing kinase (NIK) phosphorylation of IKKα dimers, and subsequent phosphorylation of p100 by IKKα [9]. This phosphorylation cascade triggers the translocation of RelB/p52 dimers into the nucleus to modulate gene transcription [14] (Figure 1).

### 1.3. Implication of NF-κB Signaling in Cancer

Considering the unique mechanism of gene regulation, NF-κB has been implicated in a diverse range of cellular processes such as inflammation, cell survival, and cell differentiation. Notably, there has been a growing amount of evidence indicating the pivotal role of NF-κB in cancer initiation and progression [15]. NF-κB is highly involved in cell proliferation via the regulation of cell cycle proteins. For instance, NF-κB was reported to trigger cyclin D1 expression in breast carcinoma cells and was found to interact with cyclin-dependent kinase 2 (CDK2) and cyclin E complex in lymphocytes [16,17]. Additionally, NF-κB has been shown to contribute to most cancer hallmarks including promoting metastasis, enabling angiogenesis, altering the tumor microenvironment, evading apoptosis, among others, in different tumor types [18]. Cytokines such as interleukin-17A (IL-17A) was shown to cause metastasis in hepatocellular carcinoma (HCC) by upregulating the levels of metalloproteinases (MMP) 2 and 9 through NF-κB induction [19]. Additionally, the constitutive activity of NF-κB in human prostate tumors was reported to be associated with the expression of key angiogenesis promoters such as vascular endothelial growth factor (VEGF), MMP 9, and interleukin-8 (IL-8) [20]. Unsurprisingly, the tumor microenvironment, which consists of various immune cells, is invariably transformed into a pro-tumorigenic microenvironment through NF-κB signaling. NF-κB activates the expression of distinct pro-inflammatory cytokines that engage in a feedback-loop to promote NF-κB dependent transcription of oncogenes [21]. Alongside enabling tumor growth, NF-κB also plays a vital role in preventing apoptosis in many cancers. For instance, inhibition of NF-κB activity triggered apoptosis in both lung cancer and colorectal cancer cell lines [22,23]. Cancer cells can evade apoptosis by upregulating a number of NF-κB-dependent anti-apoptotic genes such as B-cell lymphoma-extra-large (Bcl-xL), FLICE-inhibitory protein (FLIP), cellular inhibitor of apoptosis protein (c-IAP), and mouse double minute 2 homolog (Mdm2), a negative regulator of p53. [23,24,25,26]. p53 plays a key role in preserving the genomic integrity of a cell in response to cellular stress by activating cell cycle arrest or inducing apoptosis. Thus, NF-κB signaling has been speculated to hinder p53-induced apoptosis in response to chemotherapeutic agents used for cancer treatment [26]. Overall, the above evidence affirms the key role that NF-κB plays in various types of cancer.

## 2. General Role of Phosphorylation of NF-κB Pathway in Cancer 

The proteins involved within the NF-κB pathway is replete with the examples of post-translational modifications (PTM) that function to regulate NF-κB signaling. One such major PTM example is phosphorylation. Site-specific phosphorylation of both NF-κB and its upstream regulators in the pathway can determine the state of NF-κB activity in a cell. In the canonical pathway, the IKK complex is composed of the catalytic IKKα and IKKβ and the regulatory IKKγ/NEMO component. For example, upon stimulation with tumor necrosis factor alpha (TNFα) or IL-1, IKKβ is phosphorylated by transforming growth factor-β-activated kinase 1 (TAK1) at the serine residues in the signaling loop, activating the enzymatic activity of IKKβ [27]. 3-phosphoinositide-dependent protein kinase-1 (PDK1) was also reported to exert its cell survival function in tumor cells by phosphorylating IKKβ at serine (S) 181 [28]. Following its activation, IKKβ phosphorylates IκBα at S32 and 36, triggering IκBα proteasomal degradation [29,30] (Figure 1). Furthermore, NF-κB subunits in the canonical pathway, such as p50, p65, c-Rel, are also subjected to phosphorylation to regulate NF-κB activity [31,32,33]. On the other hand, the non-canonical pathway phosphorylation cascade begins with NIK activity on IKKα. NIK phosphorylates IKKα in its activation loop at S176, enabling its activation and recruitment to p100 for processing to p52 [34] (Figure 1). The activated IKKα subsequently mediates the phosphorylation of several serine residues on p100 at position S99, -108, -115, -123, and -82, leading to its processing by the ubiquitin-proteasome pathway [35]. The resulting p52 protein dimerizes with RelB, another subunit whose activity can be modified by phosphorylation at S472, -573, -390, and threonine (T) 103 [36]. Collectively, it is inarguable that phosphorylation plays an essential role in the regulation of NF-κB signaling.

Importantly, the distinct kinases and their phosphorylation patterns at each step of the NF-κB pathway have been associated with cancer. For example, the deletion of IKKβ decreases tumor occurrence in intestinal epithelial cells and reduced tumor growth in myeloid cells [37]. This outcome is likely associated with the lack of IκBα phosphorylation, which preludes NF-κB activation. Additionally, our group showed that upon treatment with IL-1β, p65 was found to be phosphorylated on S316, S529, and S536 in 293 cells, thereby enhancing many cellular functions of NF-κB [38]. In prostate cancer cell lines, phosphorylation of p65 at S536 led to increased expression of cytokines and chemokines that promotes tumor development [39]. Likewise, phosphorylation of T254, S529 -276, and -536 residues on p65 has also been reported to promote tumorigenesis in the bone, the breast, and the head and neck tissues [33]. Notably, the constitutive oncogenic activity of non-small cell lung cancer (NSCLC) was shown to be reinforced by the phosphorylation of S337 residue on the p50 subunit [40]. These reports strengthen the evidence for the significant role of NF-κB phosphorylation in different types of cancers. 

## 3. Phosphorylation of Novel NF-κB Activators in Cancer

In addition to phosphorylation of the well-known components, such as IKK, IκBα along the traditional NF-κB pathway as we described above, other novel NF-κB regulators, such as the recently identified NF-κB activators, the protein arginine methyltransferase 5 (PRMT5) and Y-box binding protein 1 (YBX1) can also be phosphorylated. These activators can, in turn, further regulate the NF-κB signaling, adding another complex but fascinating facet to the sophisticated regulation of NF-κB signaling. 

### 3.1. Phosphorylation of NF-κB Activator—PRMT5 in Cancer

#### 3.1.1. PRMT5, a Novel Activator of NF-κB in Cancer

PRMT5 is a member of the PRMT superfamily, a group of enzymes that transfers methyl to arginine residues of histone or non-histone proteins [41]. There are ten PRMTs identified in humans up to now, and all the PRMTs found so far are classified into four types based on the number and location of methylarginine [42]: type I PRMTs catalyze the formation of ω-NG-mono-methylarginine (MMA) and asymmetric ω-NG, NG-dimethylarginine (aDMA), including PRMT 1, 2, 3, 4, 6, and 8; type II PRMTs catalyze MMA and symmetric ω-NG, NG-dimethylarginine (sDMA), including PRMT5, PRMT7, and PRMT9; type III PRMTs catalyze only MMA, and PRMT7 sometimes exhibit the property of type III PRMTs; and type IV produces d-ω-NG-mono-methylarginine, which is only found in yeast [43]. 

As a primary type II PRMT, PRMT5 has been shown to play a crucial role in mammalian development [44]. In embryonic stem (ES) cells, PRMT5 acts through methylosome protein 50 (MEP50), methylating histone H2A arginine (R) 3 to suppress some differentiation genes, which is pivotal to mouse development [45]. It has been reported that PRMT5 is ubiquitously distributed in different human tissues and both the endoplasm and nucleus, and that it is capable of methylating a set of histone and non-histone proteins to exert various biological effects [46]. For instance, PRMT5 can regulate gene expression, DNA damage repair, mRNA splicing, proliferation, and apoptosis by methylation of histone (H4R3, H3R8, H3R2, and H2AR3) and non-histone proteins such as tumor protein 53 (p53), the p65 subunit of NF-κB, and E2F1, etc. Once the catalytic activity of PRMT5 is dysregulated due to either altered PRMT5 expression or altered expression of its target proteins and regulators that control its enzymatic activity, tumors can occur spontaneously [47]. Gao and his colleagues reported PRMT5 overexpression and mutations found in a broad range of cancers by analyzing the cBioPortal database [48]. This report indicated that PRMT5 might be an oncogene.

Importantly, a few years ago, our lab became the first to discover that PRMT5 dimethylates the p65 subunit of NF-κB at R30 to drive the activation of NF-κB signaling [49]. Our group showed that R30 mutants of p65 significantly downregulate NF-κB biological activity via decreasing the binding affinity of p65 to its target gene. We further reported that the PRMT5-NF-κB signaling axis is critical to the progression of many cancers, including colon cancer and pancreatic cancer [50,51]. Therefore, we identified PRMT5 as a novel activator of NF-κB in cancer. 

#### 3.1.2. Effect of PRMT5 Phosphorylation on NF-κB Signaling in Cancer 

Interestingly, we further revealed that the novel NF-κB activator—PRMT5—is also subjected to phosphorylation, which can modulate its activity to promote different types of cancers. Given the methyltransferase activity of PRMT5 on NF-κB activation, there is a possibility that the differential phosphorylation marks on PRMT5 may affect the tumor-promoting function of NF-κB in cancers. Recently, our lab discovered the novel phosphorylation on the S15 residue of PRMT5 [50]. We showed that phosphorylation of PRMT5 at S15 by protein kinase C iota (PKCι) enhances colorectal cancer cell growth, motility, colony formation, and anchorage-independent growth. Similarly, alteration of S15 to alanine (A) (S15A) in PRMT5 abrogated the stimulation of PRMT5 activity by IL-1β and consequently inhibits NF-κB activation and the expression of its target genes [50]. This study highlights the crucial role of phosphorylation in modulating not only NF-κB itself but also its activators, such as PRMT5, thereby indirectly regulating the NF-κB signaling. 

### 3.2. Phosphorylation of NF-κB Activator—YBX1 in Cancer

#### 3.2.1. YBX1, a Novel Activator of NF-κB 

YBX1 is a well-known DNA/RNA-binding protein that acts to regulate transcription as well as translation. Furthermore, YBX1 is known to interact with DNA and RNA, and it regulates many DNA- and mRNA-dependent processes, several of which include DNA replication, repair, and transcription, as well as DNA-dependent environmental stress, chromatin remodeling, and pre-mRNA splicing [52]. Importantly, knockdown of YBX1 can lead to embryonic lethality [53]. Unsurprisingly, due to YBX1’s critical functions in the cell, its high expression has been identified in a multitude of cancers, including breast, colon, lung, ovarian, prostate, and thyroid cancer [54,55]. 

Recently, our lab has discovered YBX1 as a novel NF-κB activator in colon cancer [56,57], where overexpression of YBX1 could promote cancerous phenotype. Additionally, YBX1 plays a vital function in renal cancer carcinomas through its interaction with secreted phosphoprotein 1 (SPP1) and Ras-GTPase activating protein SH3 domain-binding proteins 1(G3BP1), where direct protein-protein interaction can promote NF-κB activation and therefore, cancerous phenotype [58]. Moreover, YBX1 interaction with mRNA has been shown to be involved in breast cancer progression, where microRNA miR-886 was shown to bind to YBX1 and downregulate NF-κB signaling [59]. These studies and several others underly the critical importance of YBX1-mediated NF-κB signaling and the critical role of YBX1 as an activator of NF-κB.

#### 3.2.2. Effect of YBX1 Phosphorylation on NF-κB Signaling in Cancer 

PTMs on YBX1 can dynamically regulate NF-κB activity in colorectal cancers. Our lab first discovered that the phosphorylation of specific sites on YBX1 is critical for colon cancer progression, including S165 and S176. Furthermore, S176 and S165 have been shown to differentially regulate the expression of different subgroups of NF-κB target genes, thereby leading to NF-κB-mediated regulation of cancerous phenotypes [56,57]. Additionally, S102 is a widely studied residue on YBX1 that is subjected to phosphorylation, and it determines the subcellular localization of YBX1 and has crucial implications for the clinical outcome of different cancers. In breast cancer, inhibition of S102 phosphorylation by Akt activation was shown to reduce tumor growth, attenuate YBX1 nuclear translocation, and led to a decrease in antiestrogen drug resistance [60,61]. The above evidence confirms the important role that phosphorylation of YBX1 plays in cancer progression, either through NF-κB dependent or non-dependent pathways. 

## 4. Phosphorylation of Other Known Regulators of NF-κB Pathway 

In addition to PRMT5 and YBX1 phosphorylation described above, NF-κB has a range of other upstream regulators that can modulate its diverse impact on cancer progression (Table 1). The function of these regulators can be modified via phosphorylation, thereby stimulating or inhibiting NF-κB-dependent gene transcription. For example, a study discovered that cyclin-dependent kinase 4/6 (CDK4/6) phosphorylation of Rb at S249 and T252 enhances Rb interaction with p65, thereby inhibiting p65-dependent transcription of programed death-ligand 1 (PD-L1), among other genes, in metastatic prostate cancer [62]. This unique interaction, mediated by phosphorylation, highlights the role of NF-κB in regulating cell cycle and promoting evasion of the immune surveillance by tumor cells. Additionally, Deng and colleagues demonstrated that the phosphorylation of eukaryotic initiation factor 2α (eIF2α)—a key process involved in unfolded protein or endoplasmic reticulum (ER) stress response—at S51 promotes NF-κB signaling, suggesting another instance through which NF-κB activity enables cancer development [63]. Similarly, phosphorylation of mitogen- and stress-activated protein kinase 1 (MSK1) by p38 has been shown to further promote phosphorylation of p65 at S276, an important residue that aids NF-κB oncogenic function [64,65,66]. Interestingly, IKKα also plays an important role in regulating NF-κB function outside of the proteins within its pathway. For instance, IKKα-mediated phosphorylation of cyclic AMP response element binding protein (CBP) on S1382 and S1386 has been shown to increase NF-κB gene-dependent expression and promote cell proliferation in ovarian, liver, lung, and pancreatic cell lines [67]. This study also showed that this site-specific phosphorylation on CBP switches CBP association from p53 to NF-κB, further indicating the significance of phosphorylation in facilitating the survival state of a cancerous cell. In Table 1, we list some representative examples of other known regulators of NF-κB signaling that are subjected to phosphorylation.

## 5. Application of Phosphorylation of Regulators in NF-κB Signaling 

**Figure 2:**
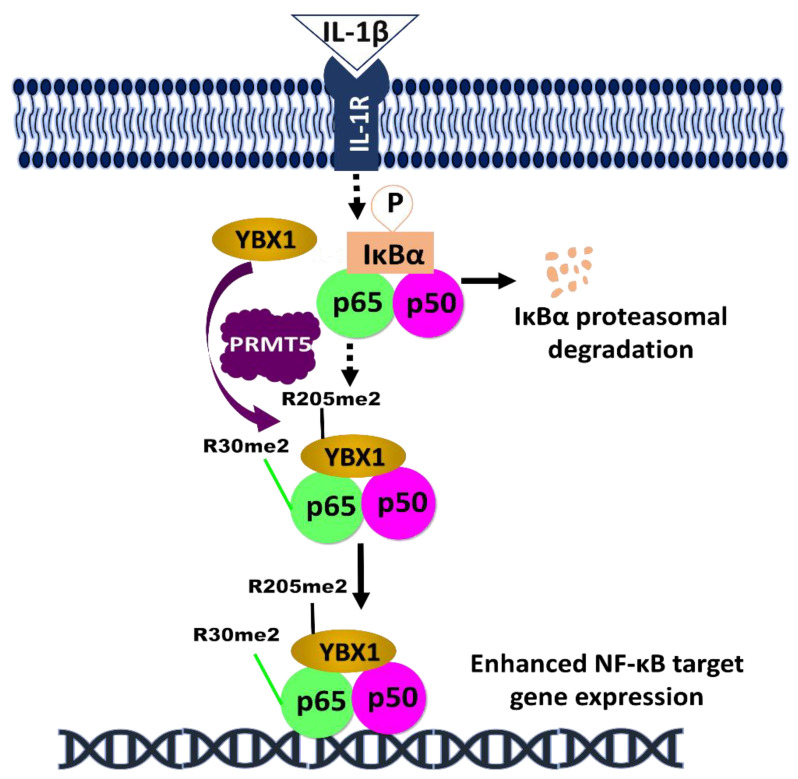
Hypothetical model of the complex regulation of NF-κB by YBX1 and PRMT5. Following the induction of NF-κB by IL-1β treatment, IKKβ is activated and phosphorylates IκBα, leading to its proteasomal degradation. This promotes the nuclear translocation of NF-κB dimers. Simultaneously, PRMT5 dimethylate YBX1 at R205 [72], and p65 at R30 [49]. On one hand, the R205me2 modification may enhance the interaction between p65 and YBX1, thereby YBX1-mediated activation of p65. On the other hand, p65-R30me2 augments the NF-κB activity and together with YBX1, to enhance the overall DNA binding capability of p65 and further promote cancer progression.

The impact of phosphorylated PRMT5 and YBX1 on NF-κB signaling in cancer, amongst other regulators, underscores the relevance of phosphorylation in aiding tumor progression. As discussed above, both PRMT5 and YBX1 serve as a potential therapeutic target for cancer treatment. Following the pioneering discovery of PRMT5 methylation of R30 residue on NF-κB by our lab [49], other studies have reported the clinical significance of the NF-κB signaling in perpetuating PRMT5 oncogenicity. In a recent study, the analysis of breast tumor tissue samples collected from 80 breast cancer patients revealed increased expression of PRMT5, which correlated with decreased levels of a tumor suppressor gene, Liver X receptor α (LXRα). Increased levels of LXRα, caused by PRMT5 inhibition, was also reported to inhibit NF-κB expression and consequently reduce cell growth, cell invasion, and glycolysis [71]. Similarly, another study showed increased YBX1 expression in 32 renal cell carcinoma (RCC) patients, and the genetic ablation of YBX1 in vitro was shown to inhibit the phosphorylation of NF-κB on S536. This pathway is crucial to RCC metastasis [58]. Interestingly, our group recently delineated the relationship between the transcription factors YBX1, NF-κB, and PRMT5 in colorectal cancer. We reported that PRMT5 dimethylates YBX1 on R205, enhancing NF-κB activation. This activation resulted in the proliferation, motility, and anchorage-dependent growth of colorectal cancer cells [72] (Figure 2).

Inhibition of kinases that mediates the phosphorylation of NF-κB regulators and NF-κB subunits may serve as a practical strategy to attenuate the aberrant NF-κB signaling identified in many different cancers. For instance, luteolin, a flavonoid emerging for its anti-cancer potential, has been identified as a p90 ribosomal S6 kinase (RSK) inhibitor by Reipas and colleagues. They further showed that luteolin exerts its anti-tumorigenic action by blocking the phosphorylation of YBX1 by RSK, leading to reduced growth of triple-negative breast cancer cells as well as its tumor-initiating cells [68]. The result of RSK inhibition is the suppression of Notch4 signaling - an important signaling pathway known to activate the NF-κB canonical pathway, leading to the induction of mammary tumorigenesis [73]. Notably, RSK can also influence NF-κB signaling via phosphorylation of IκBα on S32, suggesting both a direct and indirect effect of RSK inhibitors on NF-κB activity [74]. Inhibition of cyclin-dependent kinase 6 (CDK6) by PD332991(Palbociclib), a highly selective CDK4/6 inhibitor, reduced NF-κB-dependent gene transcription, and the genetic ablation of CDK6 was shown to markedly decrease phosphorylation of the p65 subunit of NF-κB at S536. Additionally, increased levels of CDK6 and phosphorylated p65-S536 was observed in lymphoid tumors in mice [75]. Thus, there is a possibility that the anti-tumorigenic action of Palbociclib on certain types of tumors occurs via the CDK6/p65 signaling axis. Similarly, our lab showed that the administration of PKCι inhibitor, CRT0066854, significantly reduced the expression of NF-κB target genes such as Chemokine (C-C motif) ligand 20 (CCL20) and IL-8 and decreased colon cancer cells’ growth and migration [50]. A separate study reported the discovery of a host of compounds that selectively inhibits IKKα using molecular dynamics simulations [76]. This indicates a promising path to the development of viable anti-cancer drugs that target IKKα, given its important role in regulating the function of NF-κB activators and the NF-κB non-canonical pathway (Table 1, Figure 1). Notably, the inhibition of casein kinase 2 (CKII)—a putative YBX1 kinase—by its inhibitors, CX-4945 (Silmitasertib) and K27, was shown to sensitize multiple myeloma and mantle cell lymphoma to Bortezomib treatment via downregulating the phosphorylation of p65-S529 and STAT3-S727 [77] (Table 2)**.** This finding highlights the importance of developing inhibitors that target the kinases of NF-κB and its regulators as a treatment option to combat hyper NF-κB related cancers.

## 6. Perspective and Conclusion

The evidence for the role of NF-κB in the promotion of cell survival pathways in various cancers continues to accumulate over the years. Undoubtedly, NF-κB is key to the inflammation, metastasis, angiogenesis, immune evasion, and metabolic reprograming observed in numerous cancers. Besides other control mechanisms, NF-κB-dependent gene transcription is tightly regulated by distinct PTMs, such as phosphorylation. Both the direct phosphorylation of NF-κB subunits and the phosphorylation of NF-κB regulators have been attributed to the oncogenicity of NF-κB signaling. Particularly, the dysregulation of the p65 site-specific phosphorylation through genetic alteration of kinases, epigenetic enzymes, and transcription factors has been reported to further cause aberrant NF-κB signaling. This phenomenon has been generally observed in breast cancers, renal cell carcinoma, liver cancer, ovarian cancer, hematological malignancies, etc. From cell cycle proteins to translational machinery proteins, NF-κB is regulated by a diverse set of proteins whose activities can be further modulated by distinct kinases. This multi-layered model of regulation of NF-κB illustrates the significant impact of NF-κB signaling in maintaining cellular homeostasis. 

There is a myriad of kinase inhibitors on the market and in the cancer drug discovery and development pipeline. Undoubtedly, several of these kinases have been shown to exert considerable influence on NF-κB signaling in cancers. For example, the Food and Drug Administration (FDA) approved inhibitors such as Palbociclib, which can target CDK6, a kinase that directly modifies p65 on S536 [75]. Similarly, Silmitasertib, a known inhibitor of CKII (a YBX1 kinase), is currently in clinical trials for treating sonic hedgehog (SHH) medulloblastoma patients [77]. Although inhibiting the regulators’ kinases may exert its antitumorigenic action on proteins beyond the NF-κB pathway, these examples represent the deep wiring of NF-κB signaling in multiple cellular processes that perpetuate cancer progression. The most common drawback of kinase inhibitors is the lack of specificity, as most of these inhibitors are ATP-analogues. Hence, successfully developed kinase inhibitors need to be highly selective. This can be accomplished by leveraging the minute variation in a kinase structure, among other class of kinases, as it was depicted by Anthony et al. [76]. Since the main challenge associated with directly targeting NF-κB is the potential for systemic toxicity and immunosuppression [80], one of the attractive alternative strategies to overcome these challenges is to target the modulators of NF-κB regulators, such as kinases. Ultimately, the collective implementation of various approaches and complete understanding of the role of phosphorylation in NF-κB signaling offers a promising future for the development of more effective NF-κB inhibitors for cancer treatment, and to a broader scope, other hyper NF-κB related diseases, such as inflammation, cardiovascular diseases, diabetes, and neurodegenerative diseases as well. 

## Figures and Tables

**Table 1:** Examples of commonly phosphorylated NF-κB regulators.

NF-κB Regulators	Phosphorylation Site	Kinase	Function on NF-κB Activity	Reference
PRMT5	Ser15	PKCι	Enhances NF-κB	[50]
YBX1	Ser 102 Ser165 and Ser176	RSK CKI and CKII	Enhances NF-κB	[56,57,68]
CBP	Ser1382 and Ser1386	IKKα	Enhances NF-κB	[67]
SMRT	Ser2410	IKKα	Enhances NF-κB	[69]
MSK1	Thr581 and Ser360	P38	Enhances NF-κB	[66]
TAX1BP1	Ser593 and Ser624	IKKα	Suppresses NF-κB	[70]
eIF2α	Ser51	PKR, PERK, GCN2, or HRI	Enhances NF-κB	[63]
Rb	serine-249 and threonine-252	CDK4/6	Suppresses NF-κB	[62]

SMRT: silencing mediator of retinoic acid and thyroid hormone receptor; TAX1BP1: tax1 binding protein 1.

**Table 2:** Summary of some developed compounds targeting kinases that activate NF-κB regulators.

Kinase Inhibitor	Kinase	NF-κB Regulators	Stage in Drug Development	Type of Cancer	Reference
CX-4945 (Silmitasertib) K27	CKII	YBX1	Phase I/II clinical trial Preclinical	Medulloblastoma Mantle cell lymphoma and Multiple myeloma	ClinicalTrials.gov Identifier: [NCT03904862] [77]
LY2228820 (Ralimetinib)	P38	MSK1	Phase 1b/2 clinical trial in combination with gemcitabine and carboplatin	Ovarian cancer	ClinicalTrials.gov Identifier: [NCT01663857] [78]
CRT0066854	PKCι	PRMT5	Preclinical	Colon cancer cell lines	[50]
Compound 48 (proprietary code SU909)	IKKα	p100, TAX1BP1, SMRT, CBP	Preclinical	Osteosarcoma and pancreatic cancer cell lines	[76,79]
Luteolin	RSK	YBX1	Preclinical	Triple negative breast cancer cell lines	[70]

## Data Availability

Not applicable.
